# NLRP2 Is Overexpressed in Spinal Astrocytes at the Peak of Mechanical Pain Sensitivity during Complete Freund Adjuvant-Induced Persistent Pain

**DOI:** 10.3390/ijms222111408

**Published:** 2021-10-22

**Authors:** László Ducza, Péter Szücs, Krisztina Hegedűs, Erzsébet Bakk, Andrea Gajtkó, Ildikó Wéber, Krisztina Holló

**Affiliations:** Department of Anatomy, Histology and Embryology, Faculty of Medicine, University of Debrecen, 4032 Debrecen, Hungary; ducza.laszlo@anat.med.unideb.hu (L.D.); szucs.peter@med.unideb.hu (P.S.); hegoca@anat.med.unideb.hu (K.H.); zsike@anat.med.unideb.hu (E.B.); gajtko.andrea@med.unideb.hu (A.G.); weber.ildiko@med.unideb.hu (I.W.)

**Keywords:** interleukin-1β, NLRP2, astrocyte, persistent pain, spinal cord, CFA-induced pain

## Abstract

Our earlier findings revealed that interleukin-1 receptor type-1 (IL-1R1) was overexpressed in spinal neurons, and IL-1R1-deficient mice showed significant attenuation of thermal and mechanical allodynia during the course of the Complete Freund adjuvant (CFA)-induced persistent pain model. In the present study, we found that a ligand of IL-1R1, termed interleukin-1β (IL-1β), is also significantly overexpressed at the peak of mechanical pain sensitivity in the CFA-evoked pain model. Analysis of cellular distribution and modeling using IMARIS software showed that in the lumbar spinal dorsal horn, IL-1β is significantly elevated by astrocytic expression. Maturation of IL-1β to its active form is facilitated by the formation of the multiprotein complex called inflammasome; thus, we tested the expression of NOD-like receptor proteins (NLRPs) in astrocytes. At the peak of mechanical allodynia, we found expression of the NLRP2 inflammasome sensor and its significantly elevated co-localization with the GFAP astrocytic marker, while NLRP3 was moderately present and NLRP1 showed total segregation from the astrocytic profiles. Our results indicate that peripheral CFA injection induces NLRP2 inflammasome and IL-1β expression in spinal astrocytes. The release of mature IL-1β can contribute to the maintenance of persistent pain by acting on its neuronally expressed receptor, which can lead to altered neuronal excitability.

## 1. Introduction

The treatment of chronic pain due to inflammatory diseases is a major problem in clinical practice. Chronic pain affects approximately 20% of the human population, and in many cases, the currently available treatment is not satisfactory [1].

Pathological pain can be caused by peripheral tissue injury followed by inflammation or nerve injury. In some cases, peripheral tissue or nerve injuries and inflammation evoke long-lasting changes in nociceptive-processing microcircuits [2,3,4,5,6]. It is increasingly accepted that glial cells, particularly glia activated via neuronal stimulation, are able to modulate neuronal excitability; in this way, they can influence the plastic changes during pathological pain conditions [7,8,9]. Experimental data accumulated in recent years have suggested the existence of bidirectional communication between glial cells and neurons [10,11,12,13]. Of the huge variety of substances released by activated glial cells, pro-inflammatory cytokines (e.g., interleukin-1β/IL-1β/, tumor necrosis factor-α/TNF-α/, interleukin-6/IL-6/) appear to be of special importance in the creation of neuronal hyperexcitability [14].

From the numerous molecules involved in neuron–glia bidirectional communication, in this study we focused on IL-1β, a pro-inflammatory cytokine which plays an essential role in host response to bacterial and viral infections [15,16,17], as it is also involved in several inflammatory disorders leading to the development of chronic pain [18]. Earlier studies elucidated that the interaction of IL-1R1 with its ligand IL-1β involves glial cells such as satellite cells, Schwann cells, microglia and astrocytes [19,20,21,22]. Glial cells are major contributors to neuroinflammation in the central nervous system which trigger the NF-κB and MAPK signaling pathways, resulting in the release of further inflammatory mediators [23,24]. In the central nervous system (CNS) under normal circumstances, only a low level of IL-1β is detectable, but its amount increases severalfold during neuroinflammation [25], after injury [26] and in neurodegenerative disorders [27].

Our earlier findings also support the importance of the IL-1 signaling pathway in chronic pain: we found significant attenuation of both mechanical and thermal allodynia in IL-1R1-deficient mice during the course of CFA-evoked persistent pain [28].

It is well established that in the CNS, the main source of IL-1β is activated glial cells [14,29,30]. There are two types of glial cells, namely microglia and astrocytes, which can be activated by excitatory neurotransmitters, released from nearby neurons or in the spinal cord by primary afferent fiber terminals. The neurotransmitters include adenosine triphosphate (ATP), excitatory amino acids, substance P, prostaglandins and nitric oxide, which can all influence neuron-glia cross-talk [31].

IL-1β is produced as a 31 kDa inactive form (pro-IL-1β), which is cleaved to the bioactive form by an enzymatic process [32,33]. Some studies have identified extracellular proteases to be responsible for the IL-1β cleavage [34], but most recent works have reported the role of caspase-1 in the processing of pro-IL-1β [35]. Caspase-1 itself has to be activated by the multiprotein complex termed inflammasome. Inflammasomal assembly may be activated by various stimuli, including inflammation and bacterial or viral infection, or by ATP, heat shock proteins, etc. [36]. Following caspase-1-dependent proteolytic cleavage of pro-IL-1β, its mature form is rapidly released from the cell [35,37]. Although inflammasomes were first discovered in myeloid cells, several types (e.g., NLRP1, NLRP2 and NLRP3 etc.) are known to be expressed in non-myeloid cells (e.g., in neurons and in the glial cells of the nervous system) [38].

Although a substantial amount of experimental data has recently accumulated in the field, there have still been contradictory results about the precise cellular sources and distribution of the IL-1β and the caspase-1-activating inflammasomal complexes in the CNS during pathological pain conditions. Thus, in this study, we intended to explore the expression of these markers in control conditions and their possible rearrangement in the complete Freund adjuvant (CFA)-evoked inflammatory pain model within the superficial spinal dorsal horn.

## 2. Results

### 2.1. Mechanical Pain Sensitivity of Rats Increased Significantly Following CFA Injection into the Hind Paw

**Figure 1:**
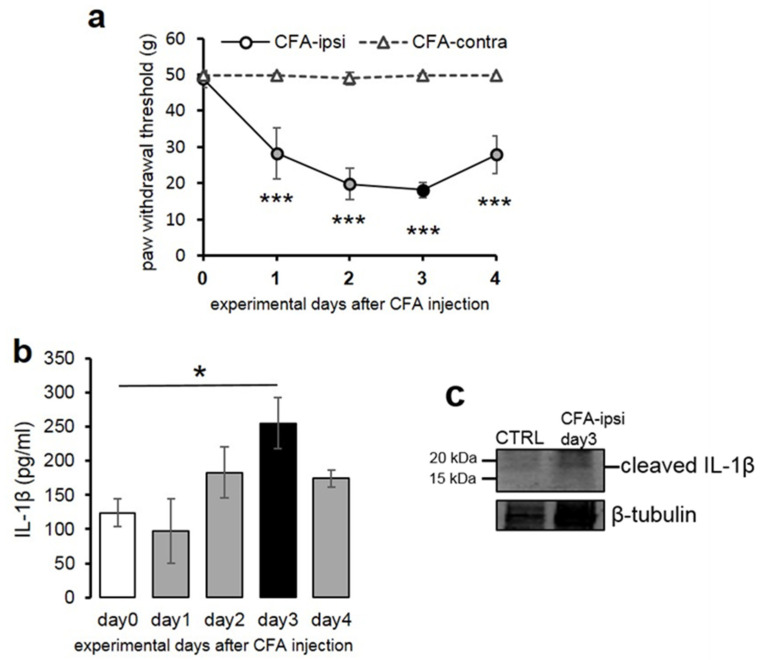
Decline of the mechanical withdrawal threshold is paralleled by the increase of spinal IL-1β production during the course of CFA-induced peripheral inflammation. (**a**) The line chart shows the mean mechanical withdrawal threshold (MWT) values on both hind limbs of animals receiving CFA injection into the right (ipsilateral) hind paw. CFA injection resulted in a substantial drop in MWT values in the ipsilateral hind paw of the treated animals, which peaked at post-injection day 3 (*p* = 0.000125). Data are presented as mean ± STD. (**b**) Bar chart shows quantitative ELISA measurement of IL-1β levels in tissue extracts obtained from the L4–L5 lumbar segments of the spinal dorsal horn of the control (day 0) and CFA-injected animals at post-injection day 1–4. Data are shown as mean ± SEM. Note that the cytokine level reached significant elevation at post-injection day 3 (*p* = 0.04). (**c**) Representative immuno-blot showing that the mature, cleaved IL-1β reached the detection level in the tissue extract at post-injection day 3. * *p* < 0.5, *** *p* < 0.001.

The mechanical withdrawal threshold (MWT) of the control animals was very similar in all animals on each of the 4 post-injection days tested, and there were no significant differences between the values measured on the left and right hind paws (data not shown). The contralateral (left, non-injected) hind paw of the CFA-treated animals also maintained the same mean MWT (48.86 ± 0.45 g). However, on the ipsi-lateral (right, CFA injected) hind paw, CFA injection resulted in a gradual drop in MWT during the first 3 days. The decline in MWT values was largest on day 3, when MWT values decreased to 23.64 ± 2.49 g (Figure 1a).

The difference between the figures of the MWT values obtained from control and CFA-treated rats was highly significant (*p* = 0.000125). Our observation that the largest MWT drop occurred on post-injection day 3 is in good agreement with the results of other authors [39,40].

### 2.2. Peripheral Inflammation Evoked by CFA Injection Induced Elevation of Spinal IL-1β Expression

Although the expression of IL-1β has already been demonstrated in the spinal cord [41], there have been no data in the literature to follow the time-dependent changes in the expression of this cytokine in inflammatory pain in rats. Thus, we intended to explore how the expression of IL-1β changes in CFA-induced inflammatory pain at the protein level (Figure 1b) in the spinal dorsal horn tissue extract of the L4–L5 spinal segments, which is known to receive primary afferent inputs from the plantar surface of the hind paw [42].

The basal level of the cytokine was 124 ± 20.05 pg/mL in the spinal dorsal horn tissue extract of the L4–L5 spinal segments, which significantly (*p* = 0.040) increased to 255 ± 37.4 pg/mL on experimental day 3, correlating with the peak of mechanical sensitivity of the CFA-injected animals. We followed the cytokine level for an additional day and observed that on day 4 of the experiment, the IL-1β concentration dropped to 174 ± 12.03 pg/mL.

**Figure 2:**
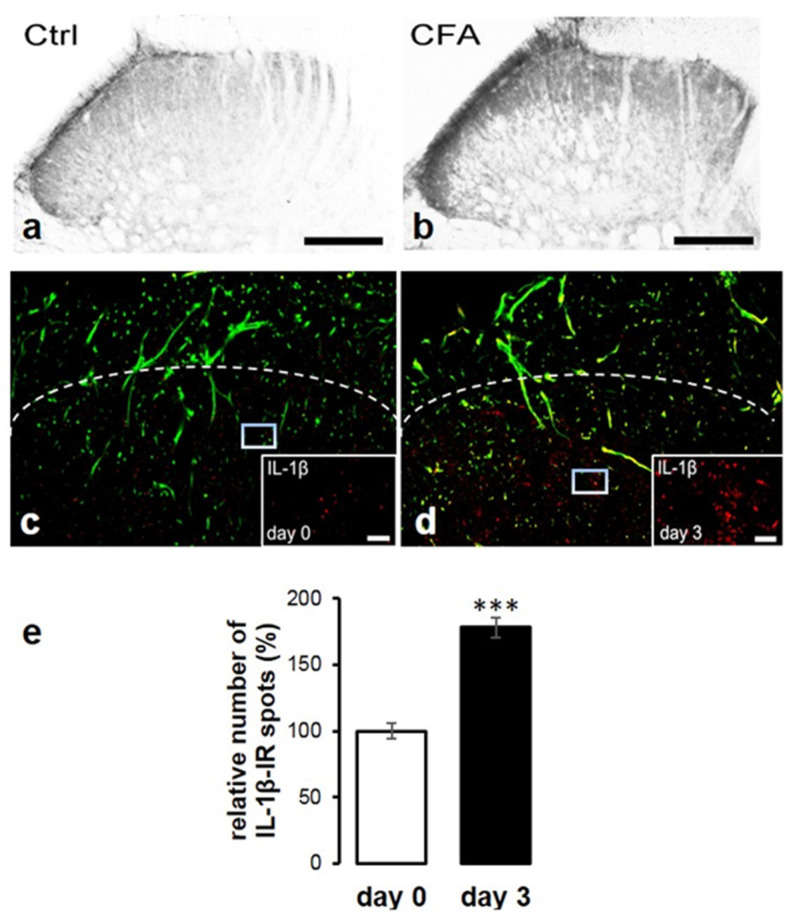
CFA-evoked inflammation of the hind paw initiates overproduction of IL-1β protein in the superficial laminae of the spinal dorsal horn of rats. (**a**) In control animals (Ctrl), a moderate immunoperoxidase reaction of IL-1β was observed in the superficial spinal dorsal horn, while almost no immunolabelling was present in deeper laminae. (**b**) In CFA-administered animals (CFA), a substantially higher immunoreactivity was detected. Scale bars: 200 μm. (**c**,**d**) Representative confocal images from superficial spinal dorsal horn show increased number of IL-1β immunoreactive puncta (red) upon CFA injection (day 3). Dashed line indicates the transition between the spinal white and gray matter. Green color shows GFAP labelling. Scale bar: 5 μm. (**e**) Quantitative analysis of confocal images showing significant elevation of IL-1β + puncta (*p* = 0.0000064) on day 3. Data are shown as mean ± SEM. *** *p* < 0.001.

As IL-1β can only bind to the ligand binding unit of its receptor (IL-1R1) if it is processed into its active form [43], we also intended to show whether the cleaved form of IL-1β is produced in the spinal dorsal horn. Western blot experiments revealed that in the control spinal cord tissue extract, the cleaved form of IL-β was present at a very low level, and its amount increased considerably on post-injection day 3 (at the peak of mechanical allodynia) (Figure 1c). Consistent with the data of Western blot and ELISA, only a moderate immunoperoxidase reaction of IL-1β was detected in the superficial spinal dorsal horn of control animals (Ctrl); however, in CFA-administered animals (CFA), a substantially higher immunoreactivity was observed (Figure 2a,b). These findings were further validated by the marked elevation of the number of IL-1β immunoreactive (IR) puncta by fluorescent immunostaining in the L4–L5 segment of superficial spinal dorsal horn of CFA-administered rats (Figure 2d—day 3) compared to control rats (Figure 2c—day 0). To provide quantitative data, IL-1β IR puncta were counted (Figure 2e); compared to the control rats, the CFA administration resulted in a significant 78 ± 7.48% (*p* = 0.0000064) increase in IL-1β production.

### 2.3. Co-Localization of IL-1β with Glial Markers

Because of its potential importance in pain processing, we investigated the expression of IL-1β on astrocytes and microglial cells by using GFAP and Iba1 as selective markers for astrocytes and microglial cells, respectively.

**Figure 3:**
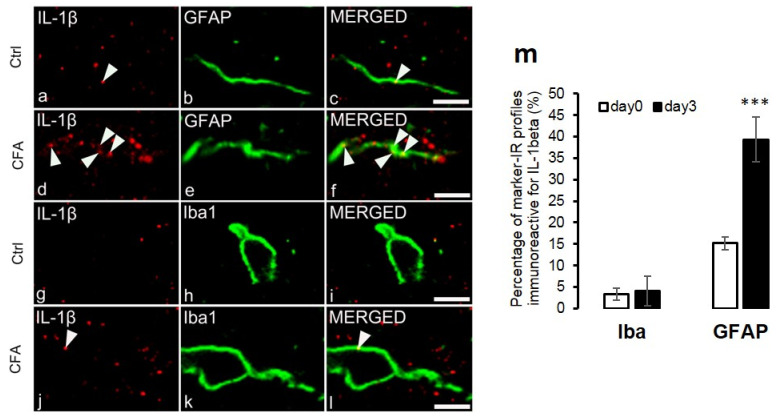
IL-1β is dominantly expressed by spinal astrocytes on post-injection day 3, at the peak of CFA-evoked inflammatory pain. The figure shows the localization of IL-1β on glial cells in the superficial spinal dorsal horn of rats. Micrographs of single 1 µm thick laser scanning confocal optical sections illustrating the co-localization of IL-1β + puncta (red) with astrocyte (GFAP, green) profiles in control conditions (**a**–**c**) and in CFA-induced inflammation (**d**–**f**). Co-localization of IL-1β+ puncta (red) with microglial profiles (Iba1, green) in control (**g**–**i**) and CFA-induced inflammatory (**j**–**l**) conditions. Arrowheads on images indicate co-localization. Scale bars: 2 μm. (**m**) Histogram showing that CFA-evoked inflammation (day 3) significantly increased (*p* = 0.002) IL-1β+ immunoreactive spots on astrocytes (GFAP) compared to control (day 0); however, IL-1β displayed minor co-localization with microglial cells (Iba1). *** *p* < 0.001.

Investigating the co-localization between IL-1β and GFAP immunoreactivity, we were looking for IL-1β-IR puncta within the confines of GFAP immunostained profiles in the control and CFA model, respectively. Analyzing IL-1β-IR spots in this location, we found that 15.15 ± 3.38% of the GFAP profiles were associated with IL-1β on sections obtained from control animals (Figure 3a–c,m). In CFA-treated animals, 39.3 ± 5.24% of the GFAP marker showed positivity for IL-1β (*p* = 0.002) (Figure 3d–f,m).

**Figure 4:**
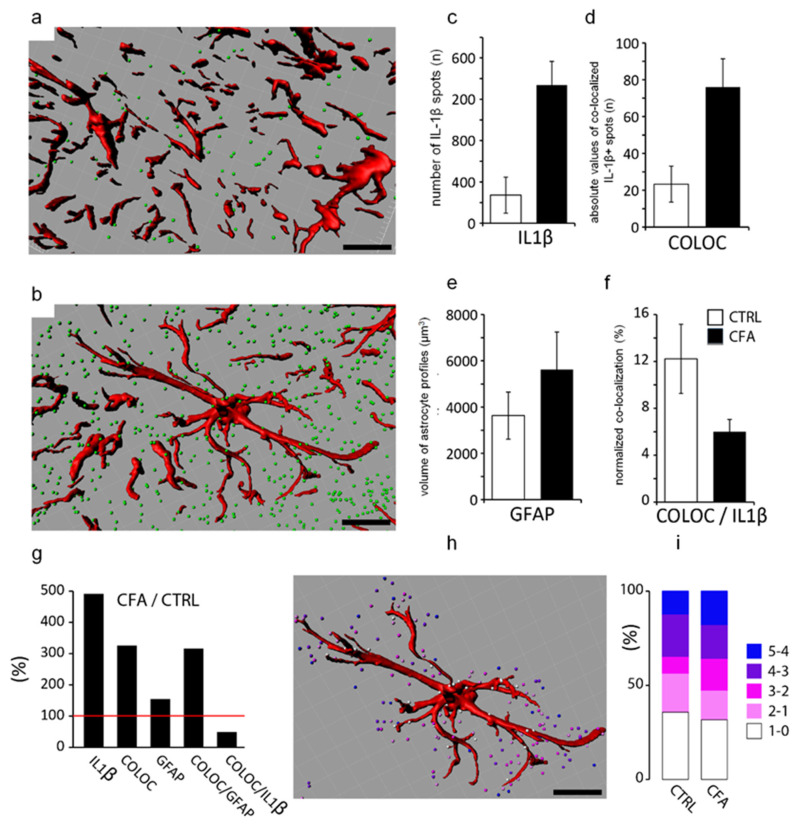
Quantitative analysis of spinal IL-1β expression and co-localization with glial markers. The co-localization with astrocytes and the morphological differences between astrocytes taken from control (**a**) or CFA-treated samples (**b**) were also quantified by IMARIS software analysis (**a**,**b**,**h**,**i**). Scale bars: 5 μm. (**c**) Software calculation showed significantly enhanced number (*n*) of IL-1β spots (*p* = 0.009032) in chronic pain (gray column) compared to control (white column). (**d**) Absolute values (*n*) of co-localized IL-1β spots (COLOC) on astrocytes were also found to be significantly higher (*p* = 0.022896) in CFA model (gray column). (**e**) IMARIS evaluation of volume change (μm^3^) in astrocyte profiles demonstrated considerable increase (gray column) (*p* = 0.194406) after CFA injection in comparison with control (white column). (**f**) Histogram showing the percentage ratio of normalized co-localization to the total number of IL-1β spots (COLOC/IL-1), indicating a significantly higher (*p* = 0.0366) co-localization in control (white column) than in CFA-treated animals (gray column). (**g**) Overall histogram shows the percentage ratio of CFA to CTRL related to IL-1β number (IL-1β), its absolute and relative co-localization with astrocytes (COLOC, COLOC/IL-1), and GFAP volumes. Data are shown as mean ± SEM. (**h**,**i**) Distance measurement (0–5 µm) of IL-1β spots from the astrocyte profiles by IMARIS program. No significant differences were observed between the control and CFA groups; the majority of IL-1β spots (%) were found within 1 μm distance from glial cells in both groups.

The co-localization between IL-1β and Iba1 was minor; only 3.31 ± 1.48% of Iba1-positive cells were immunostained for IL-1β on control sections (Figure 3g–i,m). The CFA-induced inflammatory pain condition caused a negligible elevation regarding the co-localization: 4.03 ± 1.57% of the microglial cells were observed to be positive for IL-1β. (Figure 3j–m). The characteristic morphofunctional differences between the astrocytes taken from control (Figure 4a) or CFA-treated (Figure 4b) spinal cords were also investigated by using IMARIS software. According to the calculation, the average number of IL-1β-IR spots and the absolute values of co-localized spots on astrocytes were significantly higher (*p* = 0.009032 and *p* = 0.022896, respectively) in chronic pain conditions (*n* = 1337 ± 229.2 and *n* = 76 ± 15.35, respectively) compared to control (*n* = 272.33 ± 174 and *n* = 23.33 ± 9.76, respectively) (Figure 4c,d). In addition, the volume of the GFAP-positive astrocyte profiles were also found to be substantially higher (5620 ± 1623 µm^3^), but not significantly (*p* = 0.194406) in CFA-treated samples compared to control (3630 ± 1020 µm^3^) (Figure 4e). We also performed a relative co-localization analysis, wherein the co-localization values were divided by the total number of IL-1β-IR spots (Co-localization/Total absolute number (*n*) of IL-1β-IR spots). In this analysis, a significantly higher (*p* = 0.0366) co-localization number was obtained in control (12.21% ± 2.95) compared to CFA-treated animals (5.99% ± 1.05) (Figure 4f). Data are shown as mean ± SEM.

In summary, we observed that in chronic inflammatory pain, the number of IL-1β-IR spots and the absolute value of their co-localization with astrocytes is significantly higher compared to control, which was reversed if the co-localization was normalized to the total number of IL-1β. The GFAP-positive astrocyte profiles considerably but not significantly increased in volume after CFA injection (Figure 4g). The distance of the IL-1β-IR spots from the astrocyte profiles was also measured with the IMARIS program in control and chronic pain conditions. The quantitative analysis did not reveal significant differences between the two groups; the majority of the IL-1β production was found to be within a distance of 1 µm in both cases (Figure 4h,i).

### 2.4. Distribution of NLRP Immunostaining in the Superficial Spinal Dorsal Horn

Many studies have provided evidence supporting the notion that inflammasomes play an essential role in the processing of pro-IL-1β into its active form [35]. Although it has been shown in several pain models that IL-1β is expressed in the spinal cord, we have limited knowledge about the expression of inflammasomal proteins in superficial rat spinal dorsal horn. In previous studies, NLRP1 and NLRP3 proteins were detected in the spinal dorsal horn [44,45,46,47,48,49] in neuropathic pain and spinal cord injury models. However, as of yet, there has been no attempt to explore the NLRP2 protein expression pattern in the spinal cord.

Peroxidase-based single immunostaining revealed an abundant immunoreactivity for NLRP1, NLRP2 and NLRP3 proteins throughout the L4–L5 segment of superficial spinal dorsal horn of rats, whereas the deeper laminae were more sparsely stained (Supplementary Figure S1c–f). We also detected the three investigated inflammasomal markers by Western blotting in the control spinal dorsal horn tissue extracts. All three molecules produced immunoreactive bands at the expected molecular weight (Supplementary Figure S2a–c).

### 2.5. NLRP2 Inflammasomal Protein Co-Localize with Astrocytes at the Peak of Inflammatory Pain

**Figure 5:**
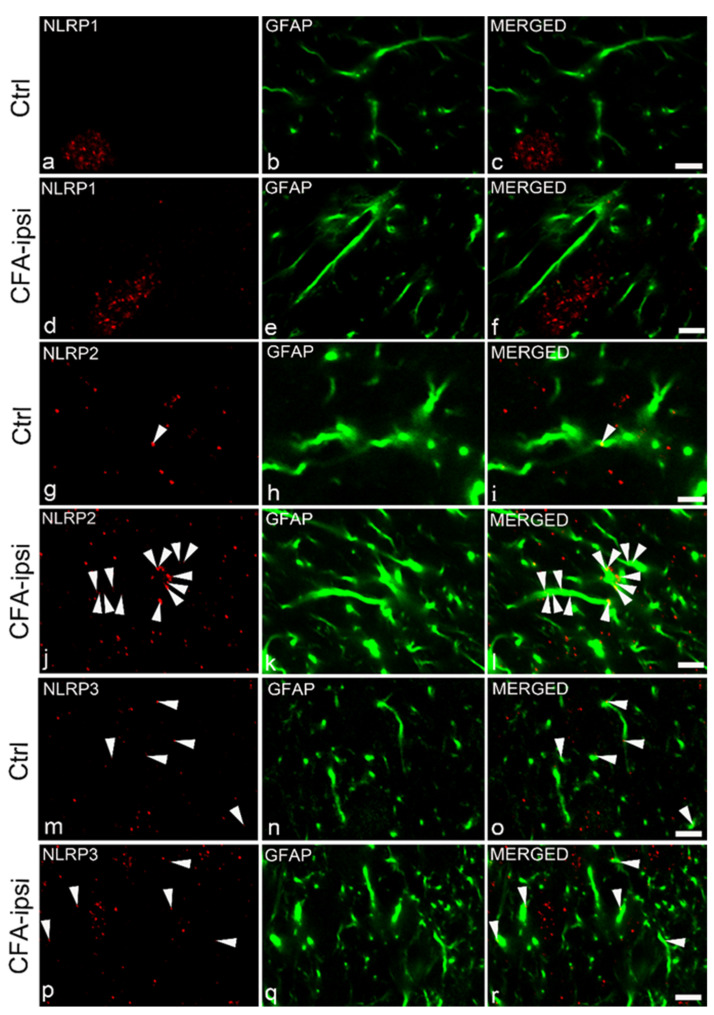
Astrocytic inflammasome expression during CFA-evoked inflammatory pain. Micrographs of single 1 μm thick laser scanning confocal optical sections illustrating the putative co-localization between immunolabeling for NLRP1 (red; **a**–**d**), NLRP2 (red; **g**–**j**), NLRP3 (red; **m**–**p**) and the immunoreactivity of astrocytes (GFAP, green; **b**,**e**,**h**,**k**,**n**,**q**) in the superficial spinal dorsal horn. Mixed colors (yellow; marked by white arrowheads) on the superimposed images (**c**,**f**,**i**,**l**,**o**,**r**) indicate double-labeled structures. For each inflammasomal marker, the first row of the images are taken from control samples (Ctrl), whereas the second row of images represents chronic inflammation (CFA-ipsi). Spinal astrocytes dominantly express NLRP2 (and to a lesser extent, NLRP3) inflammasomal markers, whilst there is a total segregation between NLRP1 and GFAP labeling. Scale bars: 5 μm.

**Figure 6:**
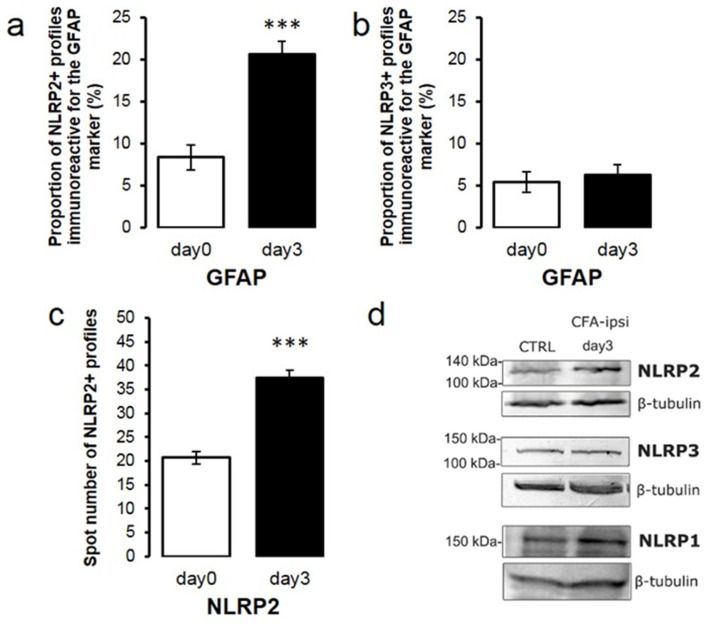
Astrocytic NLRP2 expression, but not NLRP3, is significantly increased during CFA-evoked inflammatory pain. (**a**) Histogram shows that the CFA-evoked inflammation induced an increase in the degree of co-localization between GFAP and NLRP2 markers in the superficial spinal dorsal horn of rats. Columns indicate the percentage of GFAP profiles that were found to also be labeled with NLRP2. White column shows data obtained from control animals (day 0), whereas the black column represents values found in CFA-injected animals 3 days after CFA injection into the right hind paw (day 3). CFA-evoked inflammation significantly increased the proportion of NLRP2 immunoreactive spots on astrocyte profiles (*p* = 0.000000148). Data are shown as mean ± SEM. (**b**) Histogram with the same key shows that CFA injection did not cause significant changes in the degree of co-localization between GFAP and NLRP3 markers in the superficial spinal dorsal horn of rats. Data are shown as mean ± SEM. (**c**) Histogram with the same key showing a significant enhancement of NLRP2 immunoreactive spots in chronic inflammatory pain compared to control (100%) (*p* = 0.000000111). Data are shown as mean ± SEM. (**d**) Western blot analysis shows substantial increase of NLRP2 protein in tissue lysates of spinal cord 3 days after CFA administration (CFA-ipsi day 3) compared to control (CTRL). NLRP1 and NLRP3 proteins are also detectable in the tissue samples. *** *p* < 0.001.

Considering our finding that the main sources of the IL-1β cytokine are spinal astrocytes in the CFA-evoked pain model, our next goal was to explore the astrocytic expression of the inflammasomal markers which can be responsible for the activation of caspase-1 and the consecutive maturation of IL-1β. All three investigated inflammasome types have already been reported in astrocytes [24] in different areas of the CNS; however, in the spinal dorsal horn, the NLRP1 marker showed almost total segregation from the GFAP IR profiles (Figure 5c–f). Co-localization was minor between the astrocytes and the inflammasomal marker NLRP3 (Figure 5o,r). Quantitative analysis of the fluorescent images reveals 5.36 ± 1.21% co-localization which is basically unchanged on post-injection day 3 (6.21 ± 1.29%; Figure 6b). However, 8.34 ± 1.5% of the astrocytes expressed NLRP2 protein on sections obtained from control animals (Figure 5i and Figure 6a), which significantly (*p* = 0.000000148) increased to a 20.59 ± 1.6% value on post-injection day 3 (Figure 5l and Figure 6a). (Wide field images of spinal dorsal horn are shown in Supplementary Figure S3).

### 2.6. NLRP2 Expression Is Elevated at the Peak of Mechanical Sensitivity during CFA-Induced Inflammatory Pain

Although the NLRP2 protein has already been reported to be expressed in cortical and hippocampal astrocytes [48,49], we have not found data about its distribution in the spinal dorsal horn and its possible expressional changes due to peripheral inflammation. In the preliminary experiments, we already detected the NLRP2 protein in control spinal cord tissue extracts (Supplementary Figures S1 and S2), and when we compared its basal expression with the CFA-treated samples, we could demonstrate elevation on post-injection day 3 (Figure 6d). Also, quantitative analysis of the spinal cord sections revealed that the absolute number of the NLRP2 IR puncta was considerably (*p* = 0.000000111) elevated, with 81.5 ± 7.65% compared to control (Figure 6c).

**Figure 7:**
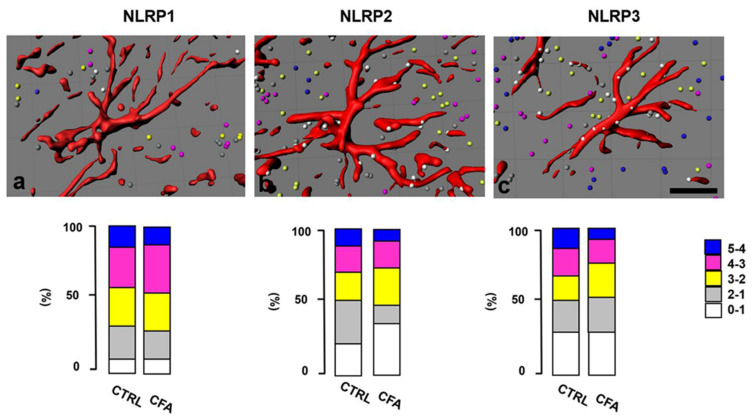
Distance measurement of NLRP markers from astrocyte profiles. Illustrations showing IMARIS rendered confocal double-stained z-stack images with putative distance (0–5µm) of NLRP1 (**a**), NLRP2 (**b**) and NLRP3 (**c**) inflammasomal proteins (colorful spots, indicated by a color scale) from GFAP-positive astrocyte profiles (red). In the case of NLRP1 and NLRP3, no significant changes were detected in distance distribution between control (CTRL) and chronic inflammatory pain (CFA); however, for the NLRP2 marker, a significant increase (*p* = 0.0004) in spot number was calculated in the range of 0–1 µm in chronic pain in comparison with control. Scale bars: 10 μm.

In accordance with the results obtained with the IL-1β distance matrix, IMARIS analysis was also carried out by calculating the distance of the three NLRP markers from astrocyte profiles rendered from confocal double-stained z-stack images (Figure 7a–c). In the case of NLRP1 and NLRP3 markers, significant changes were not found in the distance distribution between control (CTRL) and persistent pain (CFA); however, for the NLRP2 marker, a significant increase (*p* = 0.0004) in spot number was counted in the range of 0–1 µm for the inflammatory condition.

## 3. Discussion

In summary, in the current study, we investigated the expression of the pro-inflammatory cytokine IL-1β, as well as the inflammasome types which can lead to the activation of the cytokine in the spinal dorsal horn of Wistar rats during the course of CFA-evoked persistent pain. We detected that peripheral inflammation significantly increased the level of the IL-1β protein in the spinal cord on post-injection day 3, when mechanical pain sensitivity was highest. When analyzing the glial distribution of IL-1β, we found that the major source of the cytokine is spinal astrocytes, and that the astrocytic IL-1β expression is significantly increased at the peak of mechanical hypersensitivity, while spinal microglial cells provided only a minor contribution at this time point. Furthermore, we are the first to show that at the peak of CFA-induced pain in spinal dorsal horn astrocytes, the NLRP2 inflammasomal marker is overexpressed; thus, it could be responsible for the activation of caspase-1 and, consequently, for the cleavage of IL-1β and the production of the bioactive form of the cytokine.

### 3.1. IL-1β Is Produced Dominantly by Spinal Astrocytes during CFA-Evoked Inflammatory Pain

In our previous study, we observed that during inflammatory pain, the ligand-binding subunit of the IL-1 receptor (IL-1R1) is significantly upregulated on neurons in the spinal dorsal horn [28]. This finding suggested that the major target of IL-1β is spinal neurons in which the cytokine can modulate ion channel functions such as AMPA or NMDA receptors [50,51]. In the current study, our aim was to identify the cellular source of the ligand and its possible upregulation in the spinal dorsal horn. While there is much data regarding the overexpression of IL-1β during chronic pain [30,52], we intended to confirm the phenomenon by following the time course of the cytokine expression and found a correlation with the mechanical pain sensitivity levels. In our previous study [28], both mechanical and thermal nociceptive sensitivity of C57/BL6 wild type and IL-1R1-deficient mice were measured and compared to assess the effect of IL-1R1 signaling on pain sensation. However, in the current study, our objective was to validate the establishment of persistent pain in rats; therefore, we applied the routinely used mechanical allodynia test before commencing other investigations.

It is mostly agreed upon that spinal microglia are the first to be activated during chronic pain [9], but there are controversial data regarding glial activation in the later phase of pathological pain. In some models of chronic pain, spinal microglia (and in others, astrocytes) were found to be activated and consequently secrete pro-inflammatory cytokines [53]. Here, we demonstrate that in the CFA-induced persistent pain model, the predominant source of IL-1β at the time of highest mechanical pain sensitivity is spinal astrocytes, which is in agreement with other authors who have reported that astrocytic activation is more prolonged than microglial activity [9]. As in our previous study [28], we found the expression of IL-1R1 on astrocytes in addition to spinal neurons; this result suggested that not only neuronal but also astrocytic activity can be enhanced by the increased secretion of IL-1β. Earlier studies showed that IL-1β induced different responses in different cell types: while IL-1β changes neuronal excitability, in astrocytes, it mainly induces the secretion of further inflammatory cytokines and chemokines [54].

### 3.2. Cleavage of Pro-IL-1β Is Facilitated by NLRP2 Inflammasome in Spinal Astrocytes at the Peak of CFA-Induced Inflammatory Pain

For the biological activity of IL-1β, the cleavage of the precursor protein (pro-IL-1β) is essential, as only the cleaved form can bind to its receptor [55]. In most cases, the bioactive form of IL-1β is produced by the activity of the caspase-1 enzyme, which is also produced in an inactive form and requires cleavage by the inflammasomal protein complex.

Most data regarding inflammasomal expression in the spinal cord are available in neuropathic pain states, but they present a controversial picture. Among others, inflammasomes with NLRP1, NLRP2 and NLRP3 sensors are considered to be involved in the processing of IL-1β in the CNS. NLRP1 and NLRP3 have already been shown to be associated with chronic pain and expressed by astrocytes and microglia [44,45,46,47]. In addition to the two just mentioned, we also included NLRP2 in the study, as it has been reported to be expressed by human cortical astrocytes [48]. In this study, Minkiewicz and colleagues also showed that the NLRP2 inflammasome is fully functional in cultured astrocytes.

The currently available literature on NLRP2 expression and its activation is relatively limited, and several points are still unclear. Some works focus on the role of NLRP2 in the reproductive system, in embryonic development and in ischemic stroke. It has been reported that NLRP2 is connected with the development of arsenic-induced skin lesions, chromosomal damage and respiratory diseases [56]. Other authors have found NLRP2 to be associated with idiopathic recurrent miscarriage [57]. Peng et al. showed that NLRP2 is required for early embryonic development in mice [58]. However, studies on the distribution and roles of NLRP2 in CNS are very few. Cheon et al. found that in ischemic brain injury, activated astrocytes show increased expression of NLRP2 inflammasome components in the cortex and striatum, as well as in cultured astrocytes upon oxygen–glucose deprivation and reperfusion injury [59]. Sun et al. reported that the NLRP2 protein had a basal level of expression in the CNS, mainly in astrocytes, and was significantly elevated in ischemic brains [60]. A recent study by Zhang et al. detected NLRP2 in hippocampal astrocytes and found a correlation between the overexpression of the protein and depressive behavior in a mouse model of depression [49]. Until now, only one study connected NLRP2 expression to chronic pain. Matsuoka et al. showed NLRP2 expression in dorsal root ganglion cells and its activation upon tissue inflammation [61].

The NLRP2 inflammasome has already been shown to be activated by ATP [48] or TNF-α [62] stimulation; however, its specific activating signal (if it exists) has still not been revealed. An interesting feature of the NLRP2 inflammasome is that it has been shown to attenuate NF-κB activity macrophages [62], trophoblasts [63] and glioblastoma cell lines [64]. As the NF-κB signaling pathway is responsible for the production of many inflammatory mediators, the overexpression of NLRP2 can be a factor which may lead to the downregulation of the NF-κB mediated signaling pathway, which in turn can limit the production of further mediators which contribute to the maintenance of chronic pain.

However, the cellular distribution of NLRP2 in the CNS and its relation to neurological disorders such as pathological pain and cerebral ischemia still need to be further explored.

Altogether, the data presented here show that when inflammatory pain is fully developed, at the peak of mechanical pain sensitivity associated with loading-induced nociception, spinal astrocytes are activated, and their activation can significantly increase peripheral inflammation-associated nociception by the release of mediators such as IL-1β. Therefore, these results suggest that central glial activation associated with peripheral inflammation plays an important role in nociception associated with inflammatory pain.

## 4. Materials and Methods

### 4.1. Animals

The study protocol was reviewed and approved by the recommendations of the Animal Care Committee of the University of Debrecen, Hungary, according to national laws and European Union regulations (European Communities Council Directive of 24 November 1986 (86/609/EEC)], and was properly conducted according to the University’s Guidelines for Animal Experimentation. All animals were kept under standard conditions with chow and water ad libitum. The experiments were performed on male Wistar–Kyoto rats (Gödöllő, Hungary). The animals were divided into experimental groups: experimental group 1 (12 control rats) and experimental group 2 (21 CFA-treated animals). In animals in the treated group, chronic inflammation was induced by intra-plantar injection of 100 µL 1:1 mixture of physiological saline solution and CFA (Sigma, St Louis, MO, USA) into the right hind paw, according to the method described earlier [65].

### 4.2. Nociceptive Behavioral Test

Mechanical paw withdrawal threshold of the rats was tested by modified (electronic) von Frey test (Dynamic Plantar Aesthesiometer, Ugo Basile, Gemonio, Italy). Animals were placed into a cage with acrylic sidewalls and a perforated metal platform. After 15 min of habituation, a von Frey-type filament (diameter = 0.5 mm) exerted increasing force on the plantar surface of the hind paw, until the animal withdrew it. The mechanical withdrawal thresholds (MWT) for both hind paws were recorded automatically before CFA injection, then repeated daily following CFA injection. The test was repeated five times for each paw with 2 min intervals alternating between the right and the left paw. From the experimental data, mean values and standard deviations (STD) were retrieved. Statistical differences of the data were obtained by using the Kruskal–Wallis test.

### 4.3. Immunohistochemistry

#### 4.3.1. Tissue Preparation

Immunohistochemical experiments were conducted on 6 control and 6 CFA-treated adult male Wistar rats weighing 250–300 g. Three days following CFA injection into the right hind paw, intraperitoneally administered sodium pentobarbital (50 mg/kg) was applied for anesthesia. Transcardial perfusion was performed by using oxygenated physiological salt solution (mixture of 95% O_2_, 5% CO_2_) supplemented by a fixative containing 4% paraformaldehyde (for peroxidase-based immunohistochemistry and fluorescent double immunostaining). Thereafter, the L4–L5 segments of the spinal cord were removed and postfixed for 4–5 h and then rinsed in 0.1 M PB solution with 10 and 20% sucrose concentration. To ensure proper penetration of reagents, the spinal cord was immersed in liquid nitrogen and sectioned at 50 µm thickness with vibratome.

#### 4.3.2. Single Immunostaining

Laminar expression and distribution of the IL-1β, NLRP1, NLRP2 and NLRP3 inflammasomal proteins were studied by the single immunostaining method within rat spinal dorsal horns. The sections were gently shaken in PBS containing 10% normal goat serum (Vector Labs, Burlingame, CA, USA) for 50 min prior to antibody treatments. Free-floating sections were further incubated in polyclonal rabbit anti-IL-1beta antibody (diluted 1:500; PeproTech, Cranbury, NJ, USA; catalog No. 500-P80), polyclonal rabbit anti-NLRP1 antibody (diluted 1:20; Abcam, Cambridge, UK; catalog No. ab3683), polyclonal rabbit anti-NLRP2 (diluted 1:1000, Abcam, catalog No. ab36850) or mouse anti-NLRP2 antibody (diluted 1:1000; Biotechne, Abingdon, UK; catalog No. MAB4684), and polyclonal rabbit anti-NLRP3 antibody (diluted 1:1000, Abcam, catalog No. ab214185) for 48 h at 4 °C, then placed into biotinylated goat anti-rabbit IgG solution (diluted 1:200, Vector Labs) for 4 h at room temperature. Subsequently, avidin-biotinylated horseradish peroxidase complex (diluted 1:100, Vector Labs) was transferred onto the sections for 24 h at 4 °C; the chromogen reaction was visualized later with 3,3′-diaminobenzidine reagent (Sigma, St Louis, MO, USA). Following the washing steps and dehydration, the sections were firmly fixed on glass slides with DPX medium (Sigma). Images were provided by an Olympus CX-31 epifluorescent microscope.

#### 4.3.3. Double Immunostaining

Double fluorescent immunolabelings were carried out to determine the co-localization of IL-1β and NLRP proteins with other markers. Before antibody treatments, tissue sections were kept in 10% normal goat serum (Vector Labs) dissolved in PBS for 50 min, then incubated with a selection of several antibodies that contained either (a) rabbit anti-IL-1β, (b) rabbit anti-NLRP1, (c) rabbit anti-NLRP2 (d) rabbit anti-NLRP3 and one of the following antibodies: (e) mouse anti-glial fibrillary acidic protein (GFAP) (diluted 1:500; Chemicon, Temecula, CA, USA; catalog no. MAB3402), (f) guinea-pig anti Iba1 (diluted 1:2000; Synaptic Systems, Goettingen, Germany; catalog No. 234-004). Sections were gently shaken in the primary antibody solutions for 2 days at 4 °C and were further placed into the proper combination of secondary antibodies for 2 h selected from the following: (a) goat anti-rabbit IgG conjugated with Alexa Fluor 488 (diluted 1:1000; Thermo Fisher Scientific, Waltham, MA, USA; catalog No. A11034), (b) goat anti-mouse IgG-Alexa Fluor 555 (diluted 1:1000, Thermo Fisher Scientific, catalog no. A21422), (c) goat anti-guinea-pig IgG-Alexa Fluor 555 (diluted 1:1000, Thermo Fisher Scientific, catalog No. A21435). Sections were covered with mounting medium and Vectashield (Vector Labs) on glass slides.

#### 4.3.4. Confocal Microscopy and Quantitative Analysis

Single 1 µm thick optical sections were obtained with the 60x oil-immersion lens (NA: 1.4) of an Olympus FV1000 confocal laser microscope. Confocal image capturing was conducted with the same parameter settings (confocal aperture, laser aperture). Scanned images from 3 control and 3 CFA-injected animals were further processed with Olympus Fluoview 2.1 and Adobe Photoshop CS5 softwares (Olympus Corporation, Tokyo, Japan; Adobe Co., San Jose, CA, USA). Evaluation of quantitative data was based on the analysis of 3 randomly selected sections taken from each animal. The detection of the immunoreactive spots was described earlier [66]. Briefly, immunoreactive spots on the edges of grids placed on the images were counted, data collected from the 9 sections of medial and lateral areas of Rexed lamina I and II were averaged, and the values of standard error of the mean (SEM) were determined. By means of this method, control and CFA-treated animals were compared. Statistical differences between experimental groups were calculated using the Mann–Whitney test. Probabilities (*p*) of <0.05 were regarded as statistically significant.

Furthermore, certain results associated with the number and co-localization of IL-1β and NLRP markers with astrocytes were also confirmed and supplemented by using IMARIS software (Bitplane AG, Zurich, Switzerland). The spatial distribution of IL-1β immunopositive profiles was quantitatively analyzed in 12-micrometer-thick image stacks using the IMARIS (Bitplane) software. Briefly, the IL-1β positive or inflammasome sensor-positive particles were detected and GFAP-positive surfaces (i.e., astrocyte profiles) were defined by the built-in modules of the software. Next, the number of particles that fell within the boundaries of GFAP surfaces (i.e., co-localized) were counted, along with the distance of each particle (within 5 micrometers of a given GFAP-positive surface) from the closest GFAP surface point.

#### 4.3.5. Controls

The specificity of the rabbit anti-IL-1β antibody was tested on spinal cord sections by adding the diluted anti-IL-1β antibody to recombinant rat IL-1β peptide (PeproTech, catalog No. 400-01B) to reach antibody depletion. Briefly, the antibody was blended with recombinant IL-1β peptide (1 µg peptide/1 µg antibody), stored at 4 °C for 16–18 h and then centrifuged. Thereafter, the sections were first incubated with the mixture for 48 h at 4 °C and then placed into biotinylated rabbit anti-goat IgG dissolved in TPBS (diluted 1:200, Vector Labs, Burlingame, CA, USA) for 4 h at room temperature. The sections were handled equivalently as it was earlier described in the paragraph describing the single immunostaining method. The pre-adsorption of IL-1β protein to anti-IL-1β abolished the specific immunostaining (Supplementary Figure S1a). Owing to the fact that no peptides were available for testing the specificity of our antibodies against NLRP markers, secondary antibody control incubation was carried out when the primary antibody was omitted (Supplementary Figure S1b). The specific NLRP immunolabeling was abolished. In the case of the NLRP2 protein, negative and positive control immunoperoxidase reactions were also performed in lung tissue counterstained with hematoxylin staining (Supplementary Figure S1g,h).

### 4.4. IL-1β Quantitative Enzyme-Linked Immunosorbent Assay (ELISA)

A rat IL-1β/IL-1F2 Quantikine ELISA kit (RnD Systems, Minneapolis, USA; catalog no. RLB00) was utilized for the measurement of total IL-1β amount in spinal cord tissue homogenates. Briefly, control animals (*n* = 3) and CFA-treated animals (*n* = 3/day) on experimental days 1–4 were sacrificed, at which point the spinal cord was dissected and the dorsal horn of the L4–L5 segments was removed; the treated (right side) and the non-treated (left side) of the tissue were handled separately. The tissue samples were mechanically homogenized in ice-cold RIPA buffer supplemented with protease inhibitors (Pierce Protease Inhibitor Mini tablet, Thermo Scientific, Rockford, IL, USA). After 20 min of gentle rocking on ice, the samples were centrifuged (10 min, 15,000 rpm) to remove insoluble tissue debris. A 50 µL volume of supernatant was used in triplicates to determine the IL-1β content of the tissue homogenates. Then, the experiments were performed according to manufacturer instructions. From the experimental data, the mean value and standard error of mean (SEM) were calculated. Statistical differences of the data were obtained by using the ANOVA test.

### 4.5. Western Blotting

Control animals (*n* = 3) and CFA-treated animals (*n* = 3) on experimental day 3 were sacrificed; the L4–L5 segments of spinal cord tissue samples were treated similarly as for the ELISA method.

The protein concentration of the samples was measured by the detergent compatible BCA assay (Pierce, Rockford, IL, USA). The tissue homogenates were dissolved in reducing sample buffer (50 or 100 μg protein/lane) and run on 12% SDS-polyacrylamide gels (according to the Laemmli method [67]). The separated proteins were electrophoretically transferred onto PVDF membranes (Millipore, Bedford, MA, USA). The membranes were blocked with 5% normal bovine serum albumin (Sigma) in TTBS solution (20 mM TRIS, 500 mM NaCl, pH 7.5, 0.05% Tween-20). Membranes were incubated with rabbit anti-IL-1β (1:500, PeproTech), or rabbit anti-NLRP1 (1:1000, Abcam), or rabbit anti-NLRP2 (1:500, Abcam), or mouse anti-NLRP2 (Biotechne) or rabbit anti-NLRP3 (1:500, Abcam) and internal control antibody (mouse anti-β-tubulin, 1:2000, Sigma) for 2 h at room temperature. After extensive washes with TTBS, membranes were incubated with anti-rabbit Igs-HRP secondary antibody (1:1000, DakoCytomation, Glostrup, Denmark), and for the loading control, anti-mouse Igs-HRP (1:2000, DakoCytomation). The labelled protein bands were visualized with 3, 3′-diaminobenzidine (Sigma).

## 5. Conclusions

**Figure 8:**
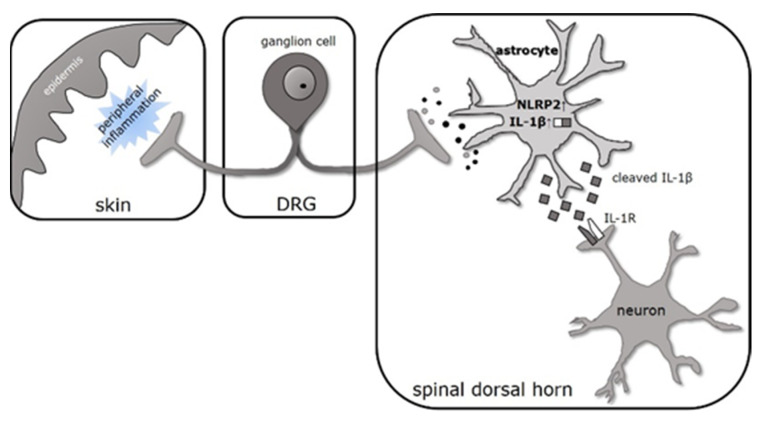
Proposed role of inflammasome activation in increased neuronal excitability during CFA-evoked persistent pain in spinal dorsal horn. CFA treatment induces peripheral inflammation in skin. Inflammatory mediators secreted by the local cells sensitize the nociceptive nerve fiber terminals and, in turn, the primary afferent fibers, terminating in the superficial laminae of the spinal dorsal horn release substances which activate astrocytes. When mechanical pain sensitivity is highest, the NLRP2 inflammasome sensor and IL-1β is overexpressed in astrocytes. Our previous findings suggest that the major target of the secreted, cleaved IL-1β is spinal neurons. IL-1R1 ligand binding affects neuronal excitability via interacting NMDA and AMPA receptor-mediated glutamate signaling, which can lead to activation of the local neuronal networks and the nociceptive sensory pathways.

The production of the bioactive form of IL-1β is under the control of multiprotein complexes called inflammasomes. Inflammasome research can provide unique perspectives for finding tissue- or cell-type-specific molecular targets for the regulation of the IL-1 signaling pathway (Figure 8).

## Data Availability

The raw data supporting the conclusions of this manuscript will be made available by the authors, without undue reservation, to any qualified researcher.

## Supplementary Materials

The following are available online at https://www.mdpi.com/article/10.3390/ijms222111408/s1.
