# Supplementary figures and images for: NLRP2 Is Overexpressed in Spinal Astrocytes at the Peak of Mechanical Pain Sensitivity during Complete Freund Adjuvant-Induced Persistent Pain
Source: Int J Mol Sci. 2021 Oct 22;22(21):11408. doi: 10.3390/ijms222111408 (PMC8584130; doi:10.3390/ijms222111408)

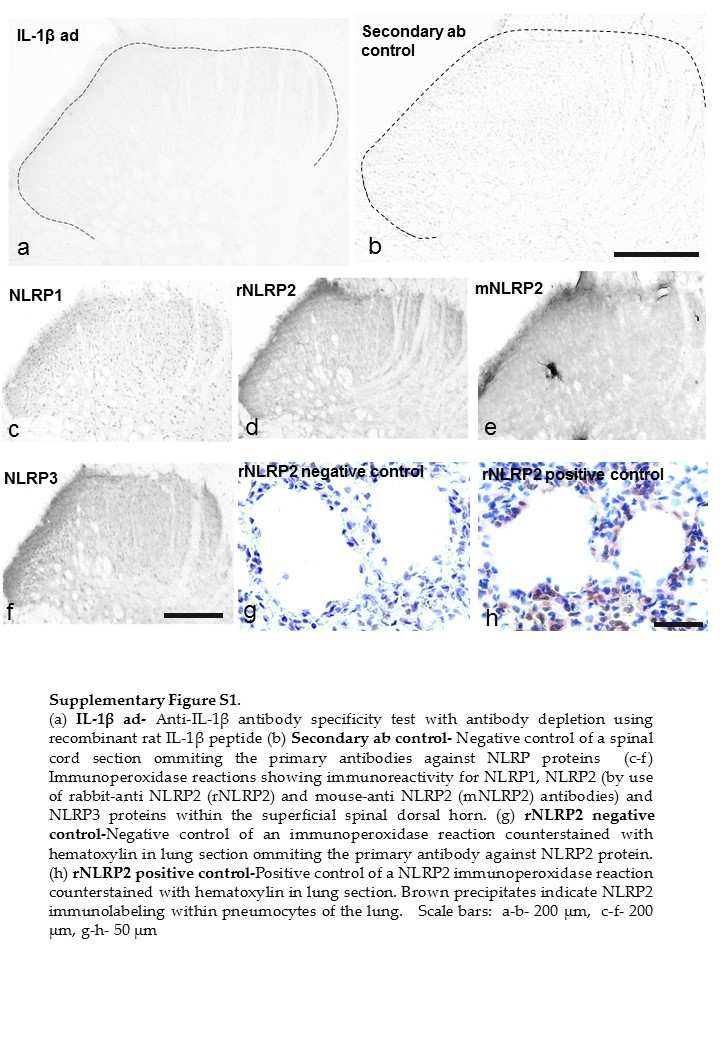

Supplement: Supplementary file 1 [file ijms-22-11408-s001.zip › SupplementaryFigureS1.JPG]

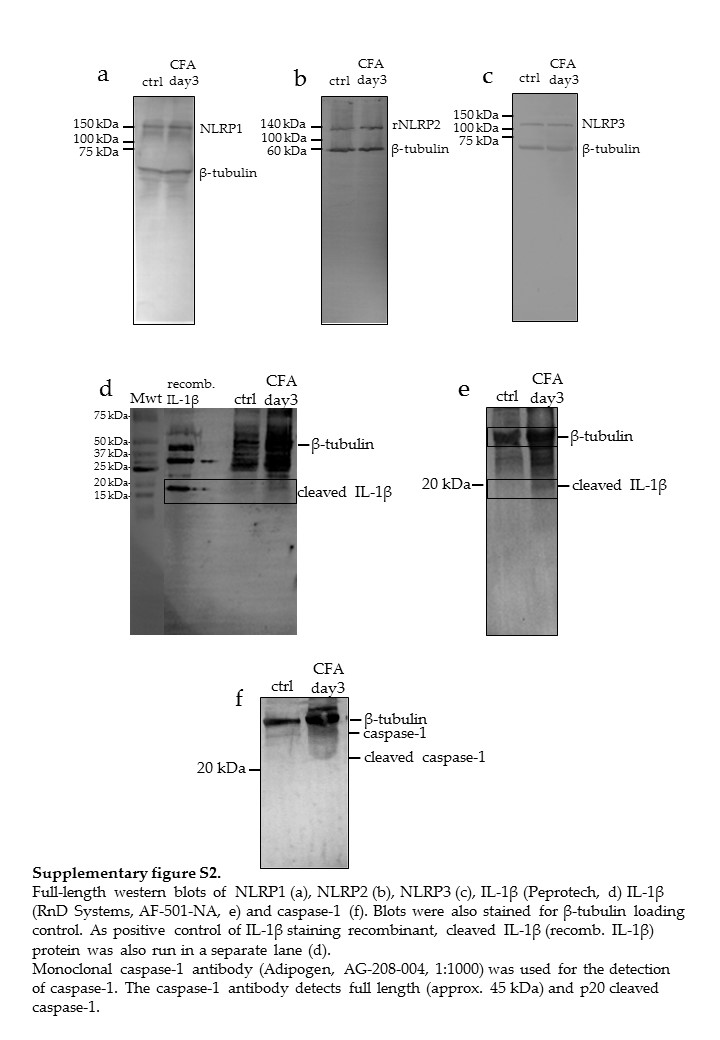

Supplement: Supplementary file 1 [file ijms-22-11408-s001.zip › SupplementaryFigureS2.JPG]

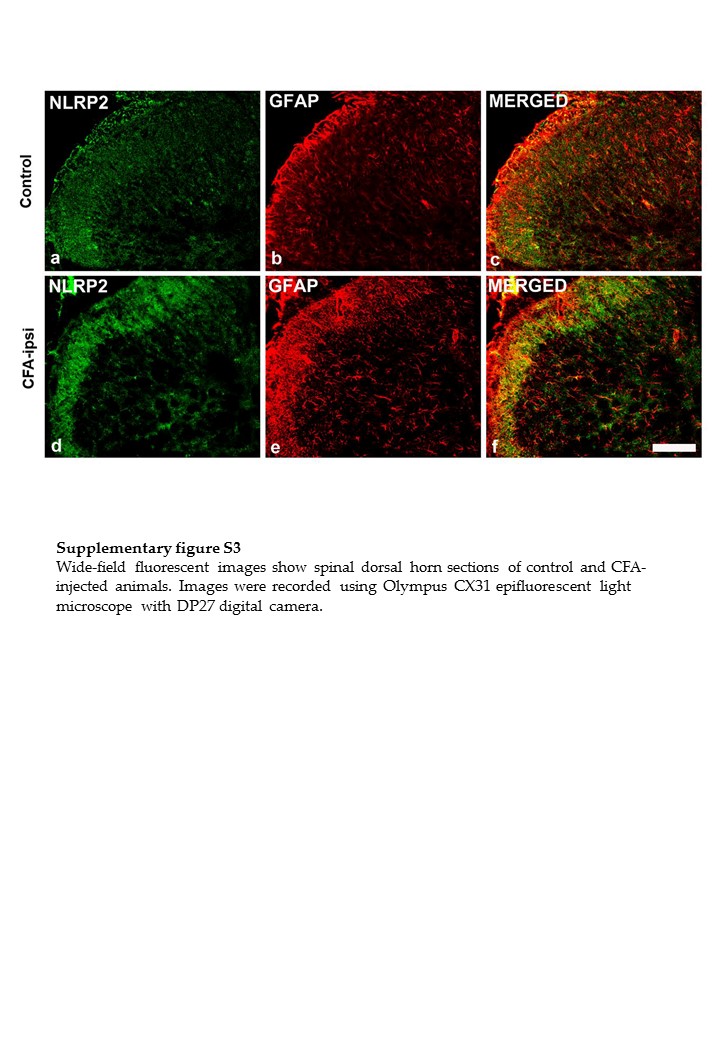

Supplement: Supplementary file 1 [file ijms-22-11408-s001.zip › SupplementaryFigureS3.JPG]
